# OR10-002 - A novel TNFR1 transcript of TRAPS gene

**DOI:** 10.1186/1546-0096-11-S1-A186

**Published:** 2013-11-08

**Authors:** C Rittore, E Sanchez, S Soler, M Albers, L Obici, MF McDermott, I Touitou, S Grandemange

**Affiliations:** 1 INSERM / CHU A.DE VILLENEUVE, Montpellier, France; 2 University Medish Centrum, Utrecht, Netherlands; 3 IRCCS Fundazion Policlinico San Matteo, Pavia, Italy; 4 6.NIHR-Leeds Musculoskeletal Biomedical Research Unit, Leeds, UK

## Introduction

Mutations in the *TNFRSF1A* gene encoding the TNF cell surface receptor, TNFR1, cause TNFR-associated periodic syndrome (TRAPS) and polymorphisms in *TNFRSF1A*, including rs4149570, rs767455 and rs1800692, are associated with inflammatory diseases.

## Objectives

We describe a novel exon 2-spliced transcript, named TNFR1-d2, and the impact of these 3 SNPs on exon 2 splicing, transcriptional activity of *TNFRSF1A* and TRAPS phenotype.

## Methods

Expression of *TNFRSF1A* transcripts was performed by RT-PCR in a range of human cells and tissues. Exon 2 splicing and transcriptional activity were analysed in HEK293T and SW480 cells by *in vitro* alternative splicing and luciferase assays, respectively. We constructed haplotypes containing rs4149570, rs767455 and rs1800692 in controls (n=70), TRAPS (n=111) and TRAPS-like patients (n=450) to compare their distribution and association with clinical features of TRAPS.

## Results

TNFR1-d2 was expressed in a tissue-specific manner, whereas TNFR1 expression was ubiquitous. Alternative splicing assays revealed that the T-A-T haplotype at rs4149570-rs767455-rs1800692 showed the highest expression of exon 2-skipping product (p=0.02). Transcriptional activity from the T-T haplotype at rs4149570-rs1800692 was increased compared to the G-C haplotype (p=0.03). In TRAPS patients, rs1800692 T/T homozygotes were excessively rare (p<10^-4^) and TRAPS-like patients with this genotype experienced less fever.

## Conclusion

Our study provides a novel mechanism of *TNFRSF1A* regulation whereby three polymorphisms in the promoter, exon 1 and intron 4 have a functional and combined effect on exon 2 splicing, via a coupling mechanism between transcription and splicing*.* These polymorphisms may impact the phenotype of TRAPS and TRAPS-like patients.

## Competing interests

None declared.

